# Analysis of Gloss Unevenness and Bidirectional Reflectance Distribution Function in Specular Reflection

**DOI:** 10.3390/jimaging10060146

**Published:** 2024-06-17

**Authors:** So Nakamura, Shinichi Inoue, Yoshinori Igarashi, Hiromi Sato, Yoko Mizokami

**Affiliations:** 1 Department of Imaging Sciences, Graduate School of Science and Engineering, Chiba University, 1-33 Yayoi-cho, Inage-ku, Chiba 263-8522, Japan; 2 Faculty of Engineering, Tokyo Polytechnic University, 1583 Iiyama, Atsugi 243-0297, Japan; sinoue@t-kougei.ac.jp; 3 Chuo Precision Industrial Co., Ltd., 65 Shirasaka Miwadai, Shirakawa 961-0835, Japan; igarashi@chuo.co.jp; 4 Graduate School of Informatics, Chiba University, 1-33 Yayoi-cho, Inage-ku, Chiba 263-8522, Japan; sato.h@chiba-u.jp

**Keywords:** material appearance, gloss unevenness, BRDF, specular reflection, measurement method

## Abstract

Gloss is associated significantly with material appearance, and observers often focus on gloss unevenness. Gloss unevenness is the intensity distribution of reflected light observed within a highlight area, that is, the variability. However, it cannot be analyzed easily because it exists only within the highlight area and varies in appearance across the reflection angles. In recent years, gloss has been analyzed in terms of the intensity of specular reflection and its angular spread, or the bidirectional reflectance distribution function (BRDF). In this study, we develop an apparatus to measure gloss unevenness that can alter the angle with an angular resolution of 0.02°. Additionally, we analyze the gloss unevenness and BRDF in terms of specular reflection. Using a high angular resolution, we measure and analyze high-gloss materials, such as mirrors and plastics, and glossy materials, such as photo-like inkjet paper and coated paper. Our results show that the magnitude of gloss unevenness is the largest at angles marginally off the center of the specular reflection angle. We discuss an approach for physically defining gloss unevenness based on the BRDF.

## 1. Introduction

**Figure 1:**
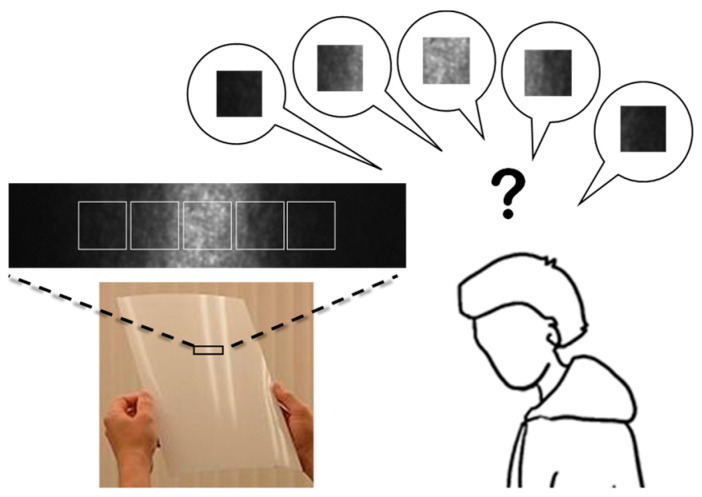
Schematic diagram showing visual inspection of gloss unevenness. In general, gloss unevenness is distributed in a narrow area near the center of specular reflection.

Gloss is an important property determining the appearance of an object. Researchers have extensively investigated human perception when observing gloss [1,2,3,4,5,6]. Meanwhile, observers often focus on gloss unevenness. Gloss unevenness is the non-uniform intensity distribution of reflected light observed within a highlight area, i.e., the microscopic variability. Many factors determine the appearance of gloss, including the distance, angle, intensity, and directivity of illumination, as well as the reflectance and roughness of the object surface. Among these factors, gloss unevenness is primarily determined by the illumination angle and the roughness of the object surface. A standardized terminology defining “gloss unevenness” does not exist, and terms such as “gloss uniformity” are used to provide the equivalent meaning [7]. A schematic diagram of the visual inspection of gloss unevenness is shown in Figure 1. In daily life, gloss unevenness may be observed in glossy papers such as those of magazines, which affects the texture appearance of the paper and the impression of the photograph. These effects are observed differently depending on whether they are presented at the strongest highlighted part or any other region within the highlighted area. Gloss unevenness is observed as a difference in shading because the incident light is reflected in slightly different directions, owing to the microscopic unevenness of the object surface. Therefore, a perfect mirror has an extremely low gloss unevenness. As shown in Figure 1, this can be described as a granular or rough texture.

Coated paper is a typical example of an object that exhibits gloss unevenness. The intensity of reflected and gloss unevenness are important factors that determine gloss [8]. Gloss unevenness can be conveniently observed by modifying the observation angle or the object orientation. However, quantifying gloss unevenness via apparatus measurements is difficult. This is because gloss unevenness is clearly observed in a narrow area near the center of the specular reflection. Second, the appearance of gloss varies depending on the reflection angle. The relationship between the angles of illumination, observation, and gloss observation is complex. When the contrast between the specular image and background is extremely low, observers are likely to assess the perceptibility of the image as an indication of gloss, whereas under contrasts, they will assess the image brightness [9]. Gloss can be evaluated based on two aspects: the distinctness of an image or the luminance difference between the specular highlight and diffuse background [10]. Paper manufacturers are constantly enhancing the quality of their products to meet consumer preferences, including addressing gloss unevenness. Evaluation technology plays a crucial role in quality improvement and will be integrated into product development processes. It is important to note that the application of evaluation technology extends beyond the paper industry.

**Figure 2:**
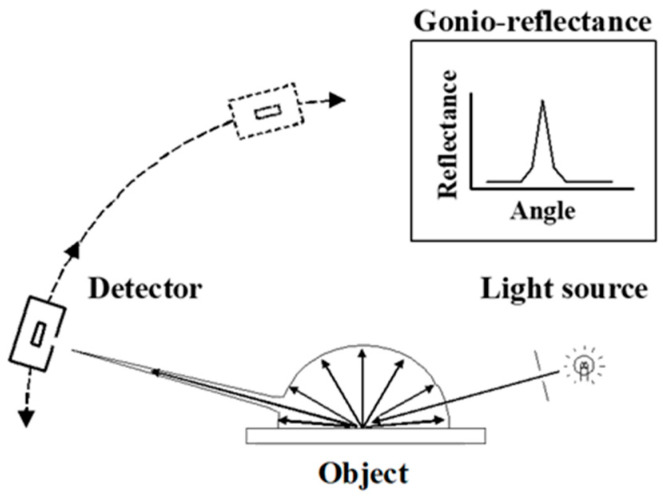
Schematic diagram of gonio-photometer and gonio-reflectance based on dichromatic reflection model.

In a dichromatic reflection model, the intensity of the reflected light is the sum of diffuse and specular reflections. As shown in Figure 2, a portion of the incident light is absorbed, scattered, and widely reflected in all directions. This phenomenon is known as diffuse reflection. The printed images exhibit diffuse reflections. Specular reflection is a mirror-like reflection of incident radiation from a surface and is particularly directional. Gloss can be quantified based on the bidirectional reflectance distribution function (BRDF). It is a function of the amount of light reflected at a particular angle when light is incident from a particular angle. The BRDF is generally used for shading in computer graphics and is useful for determining the glossy characteristics of an object [11,12,13,14].

The reflectance of each angle is known as the gonio-reflectance. It can be measured using a gonio-photometer [15]. As shown in Figure 2, a gonio-photometer features a mobile detector for measuring the reflectance at various angles under a specified incident light angle.

Reflectometry is a method for measuring BRDFs. Such methods have been proposed for various lighting environments and observational conditions [16,17,18,19,20,21,22]. Additionally, BRDFs have been analyzed by the authors [20,21,22]. However, one important issue remains to be addressed. BRDFs are typically measured at a single point within the sample instead of across an entire sample. Our intention was to measure a glossy image. Therefore, the distribution of reflected light on the surface of the sample must be determined. To accomplish this, the detection and incident light angles must be equal at all points on the sample surface.

Gloss unevenness can be measured by scanning the gloss intensity over a small area [23]. Previous authors have measured gloss unevenness using a portion of a line-light reflection image [24], which is similar to scanning the image. However, such methods are difficult to implement and require long processing times. Although researchers have attempted to quantify gloss unevenness, they have not been able to obtain detailed angular and graphical information [25,26,27,28,29,30,31]. Hence, the appearance of gloss unevenness, which depends on the reflection angle, has not been analyzed.

In this study, we develop an apparatus to measure gonio-photometric gloss unevenness and discuss a method to obtain a gloss unevenness image and the BRDF. Four materials are tested: black glass, plastic, inkjet paper, and coated paper. We further propose a method to comprehensively analyze gloss characteristics based on gloss unevenness, the peak reflection, and the BRDF, based on our measurement data. In this study, the BRDF was analyzed within a restricted range near specular reflections.

## 2. Experiment and Results

First, observation conditions for gloss unevenness were defined. Accordingly, we developed a glossy-image measurement apparatus for various incident light angles. The average intensity of the gloss unevenness image is the intensity of the reflected light on the surface. Therefore, this measurement apparatus can simultaneously measure the gloss unevenness and BRDF. Next, the gloss unevenness and BRDF were analyzed based on the measured results of the samples.

### 2.1. Observation Conditions for Gloss Unevenness

For specular reflectance measurements, the detection angles of the instrument used and angles of incident light must be equal at all points on the sample surface. However, this is not feasible when a normal light source is used because the incident light angle differs across points on the sample surface owing to the dispersion of the incident light from the light source. The gloss appearance is affected by marginal differences in the angle of the incident light. Therefore, we adopted a collimator optical system to generate incident light and a telecentric optical system to perform measurements.

**Figure 3:**
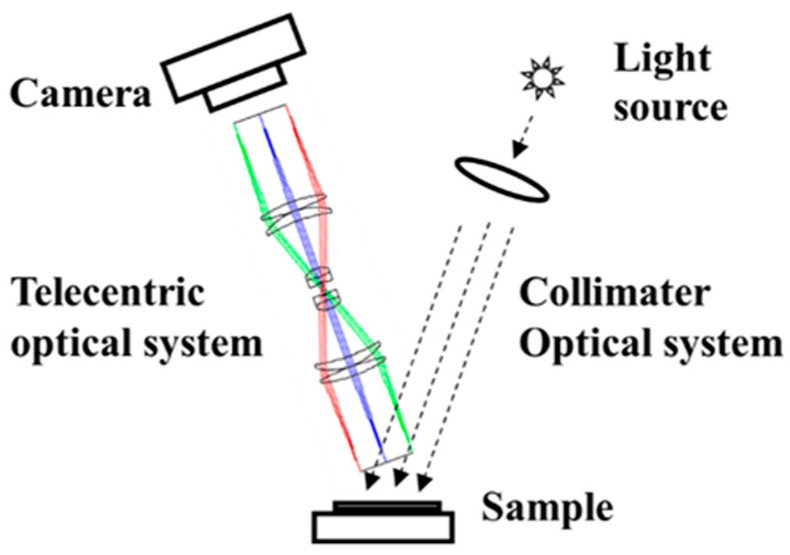
Schematic diagram of developed glossy image measurement system featuring telecentric optical system.

The incident light angle at all points on a sample surface is equal when a light source with a collimator optical system is used. Telecentric optics refers to an optical system that is parallel to the optical axis, as shown in Figure 3. The measurement angles of the reflected light at each point are equal because only the light parallel to the optical axis is measured. Our measurement apparatus employs this optical system to capture gloss unevenness images.

**Figure 4:**
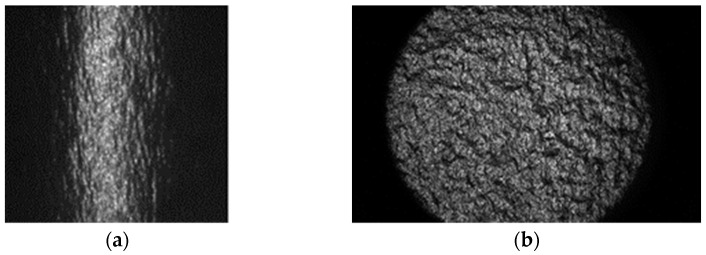
Measured gloss unevenness images using (**a**) typical lens system and (**b**) telecentric optical system. Sample used inkjet paper.

Figure 4a shows a gloss unevenness image of a linear light source captured using a normal lens and camera. The gloss dispersed around the specular reflection and the incident light angle differed depending on the point on the sample surface. By contrast, Figure 4b shows a gloss unevenness image obtained using the proposed measurement method. Because the incident light angles at all points on the sample surface were equal and only light parallel to the optical axis was measured, the specular reflection intensity at each point was measurable under the same conditions.

### 2.2. Developed Apparatus

**Figure 5:**
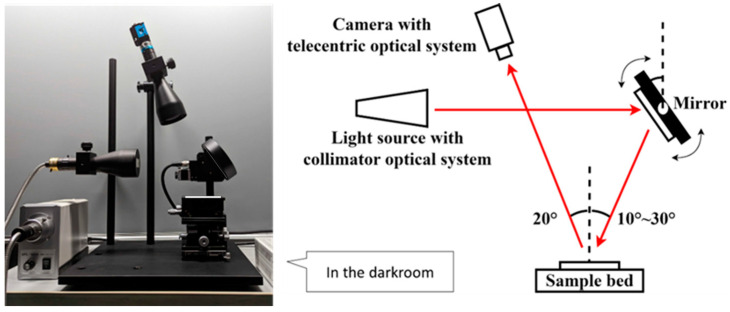
Photograph and schematic illustration of measurement apparatus, which is equipped with a collimated light source using collimator optics, a rotatable mirror, a sample bed, and a camera using telecentric optics.

The experimental setup is shown in Figure 5. The basic angle of the incident reflected light was set to 20° based on the conditions for measuring the gloss of high-gloss materials standardized by the ASTM D523 [32]. For high-gloss materials, the basic angle of reflected incident light is 20°. In this study, the incident light angle was 20.0° ± 10.0°. The measurement and camera angles for the reflected light were fixed at 20.0°. The measurement and camera angles for the reflected light were identical at all points on the sample surface. A white LED (SPL-100A, Chuo Precision Machinery, Tokyo, Japan) was used as the light source. The light emitted from the light source, which was collimated using collimator optics, was incident to a rotating mirror. The light beam had a diameter of 52 mm. The rotating mirror was rotated in 0.01° increments to vary the incident light angle on the sample. In other words, the incident light angle was varied in 0.02° increments. A CMOS camera (DMK 33UX174, Imaging Source, Bremen, Germany), which was used to measure the reflected light, was fixed at 20.0° to the sample surface. The distances from the light source to the center of the rotating mirror, from the center of the rotating mirror to the sample bed, and from the sample bed to the camera were 200, 40, and 190 mm, respectively. The image resolution of the camera was 1920 × 1200 pixels, with an output level of 12 bits per pixel. The intensity of the reflected light depended on the incident light angle. The shutter speed was adjusted to fit the measurement range of the camera. The intensity of the reflected light can be calculated using a premeasured camera calibration curve. The pitch of one pixel corresponded to 0.039 mm on the surface of the object. Because the linearity between the output value and light intensity was verified a priori, the output value can be used as the light intensity. The intensity was calculated by multiplying the pixel value by the reciprocal of the shutter speed.

### 2.3. Samples

High-gloss samples of black glass, plastic, inkjet paper, and coated paper were used in this experiment. Five types of coated papers were used, whereas one type was used for each of the other samples. A black glass plate with a sample refractive index of 1.567 was used for calibration and as a reference. Additionally, a photo-like inkjet paper was used. The standard for the coated paper was defined as the amount of pigment applied. We used coated papers manufactured under an identical standard (named A2 coated paper for printing) but produced by different manufacturers. Additionally, these samples were assumed to be isotropic and monochromatic. The intensity of the light reflected by the sample toward the detector is caused by reflection. However, a portion of this light intensity is caused by the color of the sample. In this study, these samples were assumed to be isotropic and monochromatic. Therefore, the measurements were performed in one orientation of the sample. One method for measuring gloss is specified in “Specular gloss (ASTM D523)”. This method uses the receptor field angle. A 20°/20° measurement is performed perpendicular to the measurement plane within 3.6°. Therefore, it is the integral of the amount of light reflected over a specified range of angles when the light is incident from a particular angle. Table 1 lists the specular gloss measured by ASTM D523 using a gloss meter (RhoPoint IQ-S 20/60/85, Rhopoint Instruments, Leonards, UK).

### 2.4. Measurement Results

#### 2.4.1. Gloss Unevenness Images with Different Incident Light Angles

**Figure 6:**
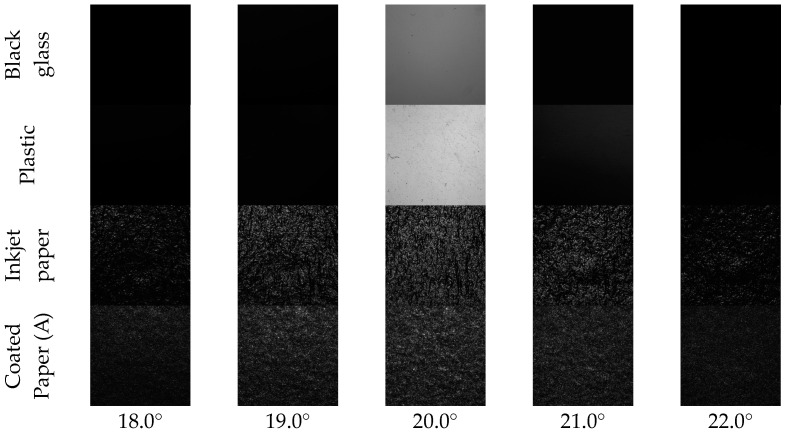
Reflected images of samples captured at five incident light angles. Black glass (**top**), plastic, inkjet paper, and coated paper (A) (**bottom**). Maximum reflectance of each sample differs significantly; hence, exposure amount (shutter speed) differs for each sample. Note that the shutter speed at each image is maintained constant even if incident-light angle changes.

The samples were measured using the developed apparatus. The images were taken at a fixed camera angle while varying the incident light angles. Figure 6 shows images of the samples captured at five incident light angles. Because the intensity of the reflected light differed depending on the sample type, the shutter speed was varied during imaging to prevent saturation. The maximum average reflected light intensity was at 20.0°, which corresponded to specular reflection. Moreover, the average reflected light intensity decreased monotonically as the incident light angle increased beyond 20.0°.

**Figure 7:**
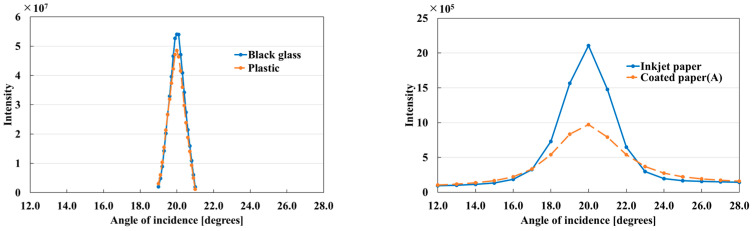
BRDFs at different angles for mirror-like (**left**) and high-gloss (**right**) samples. Horizontal and vertical axes represent incident light angle and gloss intensity, respectively. Therefore, the samples are comparable on an equal scale.

The center of the captured image was cropped, and the average pixel value for each square image was determined for each angle. The image featured 400 × 400 pixels, and the sample size was 15.6 mm × 16.6 mm. This size was selected to achieve a 2° field of view (corresponding to a diameter of approximately 14 mm at a viewing distance of 400 mm), which shall be similarly adopted in future studies for evaluating gloss unevenness. The image size of the sample was slightly larger vertically because of the camera angle. The intensity was the average pixel value multiplied by the reciprocal of the shutter speed. This calculation enabled a comparison of the samples on an equal scale. Figure 7 shows the gloss intensities at different angles obtained from the four samples. In fact, they are the BRDFs around the specular angle.

At an incident angle of 20.0°, the intensity was the highest because specular reflections were captured. The intensity decreased as the angle of incidence decreased from 20.0°. Mirror-like samples such as black glass and plastic have high peak intensities that decrease rapidly as one shifts away from the peak. By contrast, high-gloss samples such as inkjet paper and coated paper have significantly lower peak intensities than mirror-like samples (furthermore, a difference in peak intensity exists between inkjet paper and coated paper). These peaks retain their intensities even when they shift away from the peak. This is because of the strong presence of gloss unevenness in the high-gloss samples and the significant amount of reflected light, even when the incident light angle is varied.

#### 2.4.2. Definition of Gloss Unevenness

In image science, image noise is defined as the deviation in pixel values from the mean. Root mean square (RMS) granularity is a typical evaluation metric. This value is obtained by subtracting the mean of pixel values from each pixel value in an image and then calculating the standard deviation.

Another aspect must be considered when analyzing gloss unevenness. Mirror-like surfaces, such as plastic, have a high amount of reflected light, whereas high-gloss surfaces, such as printing paper, have a low amount of reflected light. Humans regulate the amount of light entering the eye through the iris and their visual sensitivity to maintain a certain amount of light within an observable range. If gloss unevenness is defined in terms of absolute light intensity, then the intensity of reflected light significantly affects the magnitude of gloss irregularities. Therefore, the dispersion of the light intensity ratio is defined as the dispersion of the light intensity ratio with gloss unevenness, as shown in Equation (1). This enables a comparison of gloss unevenness among different materials. The gloss unevenness, $GlossUnevenness\left(x,y\right)$, is defined as the ratio of the deviation from the mean value to the mean value, as shown in Equation (1):

(1) $$GlossUnevenness\left(x,y\right)=\frac{Gloss\left(x,y\right)-{Gloss}_{Mean}\left(x,y\right)}{{Gloss}_{Mean}\left(x,y\right)}$$

where $Gloss\left(x,y\right)$ is the reflected light intensity at each position, ${Gloss}_{Mean}\left(x,y\right)$ is the moving average of $Gloss\left(x,y\right)$, and x,y is the position. Gloss unevenness is evaluated based on the RMS granularity.

#### 2.4.3. Analysis of Gloss Unevenness

Gloss unevenness was observed differently at each incident light angle. We investigated the characteristics of gloss unevenness at different incident light angles. We captured a gloss unevenness image for each incident light angle. The gloss unevenness and deviation images were calculated using Equation (1). The standard deviation and RMS granularity values were calculated as well.

**Figure 8:**
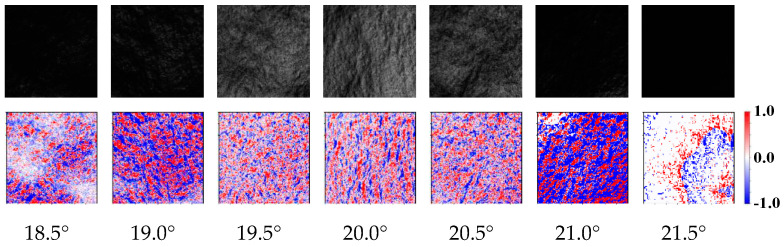
Gloss unevenness of inkjet paper at different incident light angles. In each pair of images, upper image shows captured image and lower image shows color scale. Image size was 400 × 400 pixels and sample size was 15.6 mm × 16.6 mm. Red indicates maximum deviation (1.0) and blue indicates minimum deviation (−1.0).

The result shows that the gloss unevenness differed depending on the angle. In this study, inkjet paper was selected as a representative sample. Figure 8 shows a list of gloss images and color scales. Gloss unevenness was clearly displayed on the color scale, although it could not be assessed easily from the glossy image. Dark colors on the color scale indicate coarser gloss unevenness, whereas lighter colors indicate weaker gloss unevenness.

**Figure 9:**
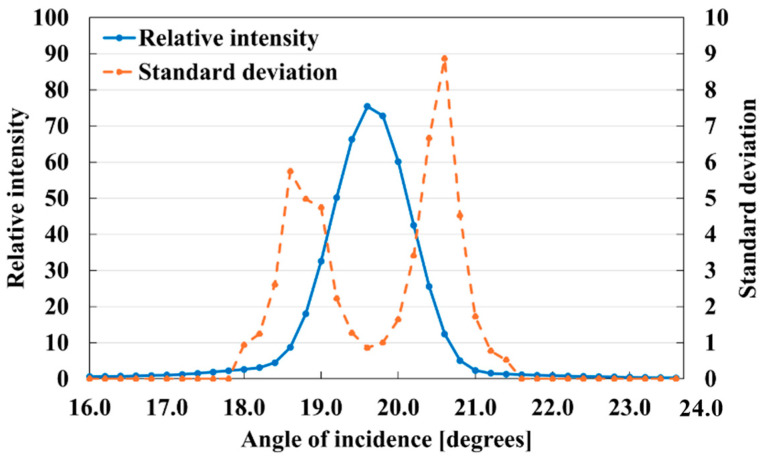
Gloss intensity and standard deviation of inkjet paper at different angles from 16.0° to 24.0° in 0.2° increments.

This was similarly reflected in the standard deviations. Therefore, the gloss unevenness degree can be approximated by comparing the standard deviations. Gloss unevenness appears to be more significant at a marginally off-incident light angle than at specular reflection. Therefore, to quantify the roughness of gloss unevenness, we calculated the standard deviation for each color scale, as shown in Figure 9. The standard deviation of the peak was approximately 1.0° relative to the specular reflection of the inkjet sample. Two peaks were clearly observed for the inkjet paper with a higher gloss.

**Figure 10:**
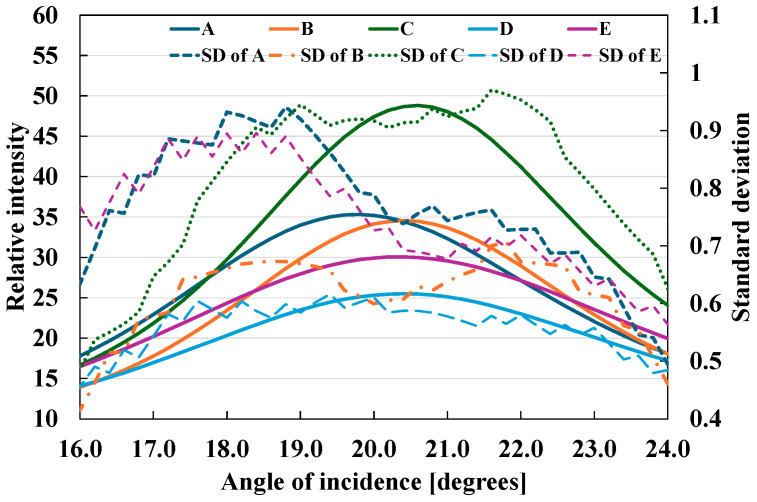
Graphs of gloss intensity and standard deviation for coated paper samples at different angles from 16.0° to 24.0° in 0.2° increments.

Subsequently, we performed this analysis on five types of coated paper that were less glossy than inkjet paper. Figure 10 shows the gloss intensities and standard deviations at different angles for the five types of coated paper. They depict the narrow-angle BRDFs around the specular reflection angle. Differences in gloss unevenness and gloss intensity distribution were observed, even for identical coated papers. The different gloss peaks likely reflected the physical inclinations of the samples. These results indicate that marginal differences can be detected, even among materials with similar specifications. In most cases, the peak of the standard deviation, i.e., the angle of maximum gloss unevenness, did not coincide with the gloss peak. Two peaks were observed for these samples.

## 3. Discussion

The developed measuring apparatus can measure uneven gloss images in 0.02° increments. The specular reflection angle range of high-gloss materials is only ±1.0°. However, we must obtain BRDF data comprising 100 uneven gloss images and 100 points in 0.02° increments. Additionally, automatic measurements using computer controls are feasible. However, the physics of gloss should be summarized using representative values. Thus, we propose a method to quantify gloss using experimental results. We will explain a method to select gloss unevenness images that can be clearly observed.

### 3.1. Gloss Quantification

**Figure 11:**
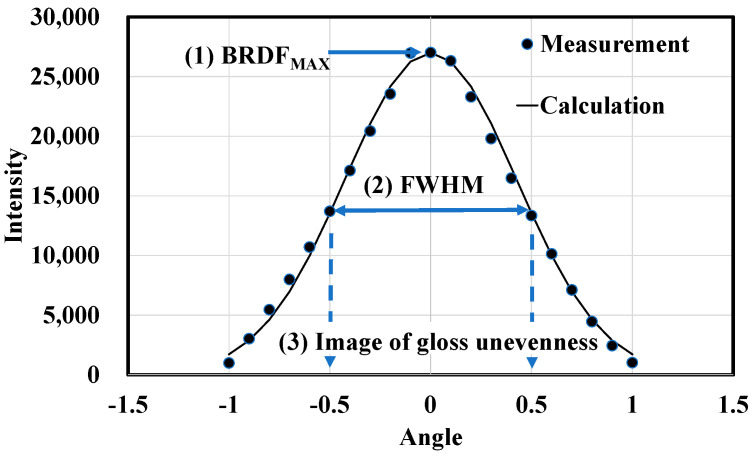
Schematic diagram of three most important points for analyzing gloss properties. Line represents calculated value using proposed model.

The three most important measurement data points for analyzing the gloss properties of a material are listed below and shown in Figure 11.

(1) BRDF_Max_: The reflection value at the peak of the specular reflection. In this study, we analyzed the phenomenon of specular reflection. Diffuse reflected light components are excluded. This is achieved by subtracting the baseline value of the BRDF curve. This is performed by subtracting the value of the base of the BRDF curve. Because the angle at which the reflected light was measured was fixed at 20.0° in this system, the reflection value was considered when the incident light angle was 20.0°. This value primarily represents the specular reflectance of an object, with high values for mirror-like samples, such as mirrors and plastics, and low values for low-gloss samples, such as paper. When comparing different sample types, one must scale the specular reflectance values.
(2) FWHM: The full width at half maximum (FWHM) of the BRDF. This value indicates the extent to which the gloss intensity decreases as the incident light angle shifts away from the BRDF_Max_. The FWHM is the full width of the incident light angle at half the BRDF_Max_. When the gloss of an object is observed using a linear light source, its center (specular reflection) is glossy. The larger the distance from the specular reflection, the weaker the gloss. In general, the higher the reflectance of the object, the more clearly the gloss is observed at the center, and the more significant the decrease in gloss. Although this system uses a collimated light source, the gloss reduction rate can be estimated by scanning the gloss intensity at each incident light angle. Our measurement data show that the BRDF curve of gloss intensity can be fitted to a normal distribution curve. Therefore, by combining the FWHM and BRDF_Max_, a BRDF mountain-shaped graph of gloss can be obtained.
(3) Image of gloss unevenness at FWHM. This image best represents the texture of the sample. The reason for using FWHM to represent gloss unevenness images is discussed in the next section. Our system can simultaneously obtain the BRDF and a gloss unevenness image at a specific incident light angle.

We propose modeling the gloss curve, i.e., the BRDF, using a normal distribution curve, which can be expressed as shown in Equation (2).

(2) $$f\left(\theta \right)=\frac{1}{\sqrt{2\pi \sigma^{2}}}exp\left(-\frac{{\left(\theta -\mu \right)}^{2}}{{2\sigma }^{2}}\right)$$

where θ is the angle of incidence, μ is the peak angle, and σ is the standard deviation of the BRDF. The relationship between σ and the *FWHM* can be expressed as follows:

(3) $$\sigma \;≅\;\frac{FWHM}{2.35}$$

The maximum value occurs on the curve when μ and θ are 0.0. Therefore, the calculated curve, $f^{\prime }\left(\theta \right)$, can be approximated using Equation (4).

(4) $$f^{\prime }\left(\theta \right)={BRDF}_{Max}*\left(\frac{{\left(\theta -\mu \right)}^{2}}{{2\left(\frac{FWHM}{2.35}\right)}^{2}}\right)$$

The calculated data are shown in Figure 11. The gloss curve can be modeled using the *BRDF*_Max_ and *FWHM*. Furthermore, the gloss unevenness can be represented by an image at the *FWHM* angle. The overall properties of the sample glosses were determined using this quantification method.

### 3.2. Gloss Unevenness Image Selection

Uneven gloss images with larger standard deviations have a rougher surface profile, which renders it more convenient to distinguish between samples when visually evaluating them. Our results indicate that an image near the peak of the standard deviation should be used to represent gloss unevenness. The results shown in Figure 9 and Figure 10 indicate that the peak of the standard deviation was located away from the specular reflection. Therefore, we propose an image at FWHM to evaluate gloss unevenness. For various samples, the FWHM was near the peak of the standard deviation and was used to model the proposed gloss curves. This facilitates its use as a unified index. Mirror-surfaced materials like black glass and plastic exhibit smooth surfaces with minimal variations in glossiness. It was observed that the standard deviation of glossiness remained consistently low, even with changes in the angle of incident light. Images of glossiness at various angles showed minimal variation. Thus, selecting the glossiness image at FWHM as a representative sample is also effective for smooth surfaces.

Figure 7 shows the BRDF of each material. The shape of the graph provides information regarding the gloss peak, gloss spread, and FWHM of the sample. The relationship between the peak intensity and FWHM is presented in Table 2.

The FWHM indicates the distance from the angle of incidence of the BRDF_Max_ to half the gloss intensity of the BRDF_Max_. For example, an FWHM of 1.0 indicates that the gloss intensity at 19.5° and 20.5° is half that of the BRDF_Max_. The BRDF_Max_ and FWHM differ significantly for the high- and low-gloss samples and are broadly classified by the sample type. Therefore, the BRDF curve can be reasonably modeled using the Max and FWHM to characterize the sample.

**Figure 12:**
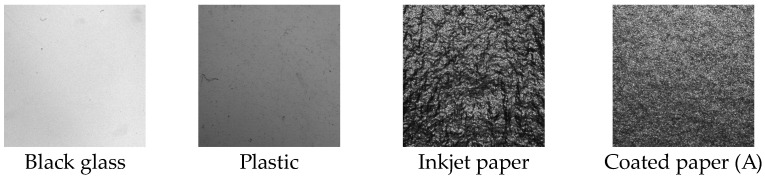
Uneven gloss images of sample at FWHM point. Image featured 400 × 400 pixels and sample measured 15.6 mm × 16.6 mm.

Figure 12 shows the cropped images of each sample at the FWHM. These images show the textures of the sample surfaces. The surface of the high-gloss sample was smooth and even, whereas that of the low-gloss sample was textured. To a certain extent, the sample type can be estimated by visually inspecting an image. If the image is near the BRDF_Max_, then the texture cannot be verified easily owing to the prominent highlights. Additionally, the images indicate the effectiveness of using a gloss unevenness image at the FWHM as a representative image. However, psychophysical examinations are necessitated because the detailed conditions under which gloss unevenness can be observed more conveniently remain unknown.

### 3.3. Apparatus Features

Our newly developed measuring device is a gonio-photometer that can obtain uneven gloss images. This implies that the BRDF of a small angular range of specular reflections can be measured. The system can be used as a gloss meter to compare the peak glosses of samples. In fact, BRDF systems that can be used as gloss meters have been investigated [33,34]. The developed device features a fixed measurement angle of 20.0°. However, if the measurement angle (and the incident light angle) varies, then measurements should be performed at the optimal angle for each sample or more detailed measurement conditions should be specified. This would likely yield better results compared with those afforded by conventional gloss meters. Most conventional BRDF measurement devices obtain the light intensity for each incident and reflected light angle. However, detailed information regarding each angle cannot be obtained. By contrast, the newly developed measurement device can capture images at each incident light angle. This enables the acquisition of detailed information regarding the object surface, such as gloss irregularities, in addition to the light intensity at a specific incident light angle from an image.

### 3.4. Applications for Flaw Detector

The developed system can acquire detailed information regarding the surface of an object, such as scratches. Thus, it can be used to detect defects in industrial products. Scratches on objects vary by intensity, angle, and material. Certain flaws can be observed in only certain directions, which hinders their detection. Using the developed system, the incident light angle can be varied in small increments to acquire images. Moreover, by integrating the information from each angle, the shapes of minute flaws can be detected.

### 3.5. Limitations of This Study

A gonio-photometer was developed in this study; however, differences exist among gonio-photometers. First, for the developed measurement device, we adopted a method that changes the illumination light angle to preserve the measured image and the sample’s surface geometry. Therefore, the same results may not be obtained when measuring the reflected light at each angle under a fixed-angle illumination light. According to Fresnel’s laws, the amount of incident radiation reflected varies across incidence angles. The luminous flux changes according to the cosine law when the angle of incident light on a surface changes. Although the error is assumed to be insignificant because it is within the angle range of specular reflections, i.e., 20°, correction is required for a more accurate measurement.

Second, the measurement results were affected by the transfer function of the optical system of the experimental device. For example, the BRDF of black glass should be similar to the delta function. Thus, the measurement device should be calibrated via comparisons with standard samples in the future for more accurate measurements.

## 4. Conclusions

We have developed a device to measure the gloss unevenness. A collimated light source and a telecentric optics camera made equalizing the incident and reflected light angles at all sample positions possible. Furthermore, a rotating mirror can control the angle of incident light. The apparatus could measure gloss unevenness by altering the angle with an angular resolution of 0.02°. This feature of measuring at gonio angles also made measuring the BRDF of specular reflection possible. We analyzed the gloss unevenness and BRDF in terms of specular reflection. Using a high angular resolution, we measured and analyzed high-gloss materials, such as mirrors and plastics, and glossy materials, such as photo-like inkjet and coated paper. The results showed that the gloss unevenness was maximum near the FWHM of the BRDF. Based on this result, we proposed a method to select an image at the FWHM of BRDF as a representative gloss unevenness image. To comprehensively determine the gloss characteristics of materials, we proposed the consideration of at least three physical quantities: the maximum intensity, the BRDF’s FWHM, and a gloss unevenness image. The results of this study are relevant to photography and human vision. Furthermore, they enable us to define gloss unevenness better. We plan to investigate the relationship between gloss unevenness and human perception in future studies.

## Figures and Tables

**Table 1:** Samples used in experiments.

Sample	Specular Gloss (ASTM D523)			Annotation
	20°/20°	60°/60°	85°/85°	
Black glass	82.0	90.5	99.0	Standard for gloss
Plastic	90.2	96.5	98.2	White acrytic resin
Inkjet paper	15.3	45.3	81.9	Photo-like inkjet paper
Coated paper(A)	7.3	36.7	84.3	A2 coated paper for printing

**Table 2:** BRDF_Max_ and FWHM for each sample.

	Black Glass	Plastic	Inkjet Paper	Coated Paper (A)
BRDF_Max_	5.40 × 10^7^	4.86 × 10^7^	21.1 × 10^5^	9.71 × 10^5^
FWHM [°]	1.0	1.0	4.0	4.0

## Data Availability

The data presented in this study are available on request from the corresponding author due privacy.
